# Draft Genome Sequences of Two Clostridium botulinum Group II Strains Carrying Phage-Like Plasmids

**DOI:** 10.1128/mra.00091-22

**Published:** 2022-05-18

**Authors:** Brigitte Cadieux, Opeyemi U. Lawal, Jean-Guillaume Emond-Rheault, Julie Jeukens, Luca Freschi, Irena Kukavica-Ibrulj, Roger C. Levesque, John W. Austin, Lawrence Goodridge

**Affiliations:** a Department of Food Science and Agricultural Chemistry, Food Safety and Quality Program, McGill University, Ste-Anne-de-Bellevue, Quebec, Canada; b Canadian Research Institute for Food Safety, Department of Food Science, University of Guelph, Guelph, Ontario, Canada; c Institut de Biologie Intégrative et des Systèmes (IBIS), Université Laval, Québec City, Québec, Canada; d Bureau of Microbial Hazards, Health Products and Food Branch, Food Directorate, Health Canada, Ottawa, Ontario, Canada; University of Maryland School of Medicine

## Abstract

Clostridium botulinum is responsible for botulism, a potentially lethal foodborne intoxication. Here, we report the draft genome sequences of C. botulinum group II strains 202F (serotype F) and Hazen (serotype E). The genomes share many similarities, including multiple mobile genetic elements.

## ANNOUNCEMENT

Clostridium botulinum is an anaerobic, Gram-positive, spore-forming bacterium that produces the neurotoxin that causes botulism (1, 2). Exposure to the neurotoxin occurs mainly through the ingestion of contaminated food but can also occur through bacterial colonization of wounds or injection of the toxin for cosmetic or therapeutic purposes (1). Based on 16S rRNA gene sequencing and their physiological differences, C. botulinum strains are divided into four groups (I to IV) (3), with most cases of foodborne botulism being associated with groups I and II (4, 5). Here, the draft genome sequences of two C. botulinum group II strains are presented.

The C. botulinum strains 202F and Hazen were originally isolated from marine sediments in California, USA, in 1965 (6), and salmon in Nova Scotia, Canada, in 1932 (7), respectively. The isolates were identified as C. botulinum and serotyped as previously described (6, 8). They were grown on a McClung-Toabe agar base containing 0.5% Bacto yeast extract (Becton, Dickinson and Company, MD, USA) and 5% egg yolk suspension for 2 to 3 days at 35°C in anaerobic conditions (9). DNA was extracted using the DNeasy blood and tissue kit (Qiagen, Hilden, Germany). DNA libraries were generated using the KAPA HyperPrep kit (Kapa Biosystems, Wilmington, USA). Paired-end (2 × 300-bp) sequencing was performed using the Illumina MiSeq instrument. The sequence reads were trimmed using Trimmomatic v0.33 (10). Reads with a per-base Phred score above 20 in at least 100 bp were assembled *de novo* using the A5 pipeline (11). Annotation was performed using the NCBI Prokaryotic Genome Annotation Pipeline v6.1 (12). Plasmids and prophages were identified using MOB-suite v3.0 (13) and PHASTER (14), respectively. The homology between sequences was determined using BLAST v2.9.0 (15). Default parameters were used for all tools.

Sequencing of strain 202F yielded 845,112 raw reads and 34 contigs with 30× median coverage, while 629,334 raw reads and 80 contigs with 25× median coverage were obtained for strain Hazen. The 202F and Hazen genomes comprise 3,829,425 bp and 3,821,401 bp, respectively, and both have 27.2% G+C content. Strain 202F contained 3,496 protein-coding sequences and 121 RNAs, while Hazen had 3,422 protein-coding sequences and 120 RNAs. A comparative analysis between the previously sequenced 202F genome (GenBank accession number CP006903.1) (2) and that obtained in this study showed 99% nucleotide sequence homology. A mobilizable plasmid (3.5 kb, contig 25) with 99% sequence homology to the *Francisella* sp. strain MA067296 plasmid (CP016929.1) was identified in 202F. Another plasmid (40.1 kb, contig 16) with 99% sequence homology to C. botulinum pCBI (CP006904.1) (2) was also present in this strain. This plasmid was identified using PHASTER as an intact phage, indicative of a putative phage-like plasmid. Additionally, two prophages (17 kb, contig 3; 28.8 kb, contig 5) were identified. For Hazen, three prophages (35.5 kb, contig 19; 39.1 kb, contig 6; 43 kb, contig 32) were identified, in addition to one probable phage (57.1 kb, contig 2) that carried essential plasmid genes (e.g., *parA*, *parB*) indicative of a putative phage-like plasmid.

### Data availability.

The genome sequences of C. botulinum strains 202F and Hazen have been deposited in DDBJ/ENA/GenBank under accession numbers NPMX00000000.1 and NPMY00000000.1 for the contigs and SRA accession numbers SRR17916278 and SRR17916277 for the raw reads, respectively.
